# Optical Fiber Vibration Sensor Using Least Mean Square Error Algorithm

**DOI:** 10.3390/s20072000

**Published:** 2020-04-02

**Authors:** Yu Wang, Jie Zou, Yuelin Xu, Yu Chen, Xin Liu, Qing Bai, Baoquan Jin

**Affiliations:** 1 College of Physics and Optoelectronics, Key Laboratory of Advanced Transducers and Intelligent Control Systems (Ministry of Education and Shanxi Province), Taiyuan University of Technology, Taiyuan 030024, China; wangyu@tyut.edu.cn (Y.W.); zoujie0676@link.tyut.edu.cn (J.Z.); liuxin01@tyut.edu.cn (X.L.); baiqing@tyut.edu.cn (Q.B.); 2 Science and Technology on Near-Surface Detection Laboratory, Wuxi 214035, China; xuyuelin700612@163.com (Y.X.); cy0520tool@163.com (Y.C.); 3 State Key Laboratory of Coal and CBM Co-Mining, Jincheng 048000, China

**Keywords:** optical fiber vibration sensor, coherent optical time domain reflectometry, semiconductor optical amplifier, least mean square error algorithm

## Abstract

In order to enhance the signal-to-noise ratio (SNR) of a distributed optical fiber vibration sensor based on coherent optical time domain reflectometry (COTDR), a high extinction ratio cascade structure of an acousto-optic modulator and semiconductor optical amplifier is applied. The prior time-frequency analysis and least mean square error algorithm are adopted in the COTDR system for amplitude demodulation and phase demodulation, in order to improve the SNR by noise elimination. The experimental results show that the adaptive filter based on the least mean square error algorithm could realize the extraction of a three-order sinusoidal harmonic signal from strong background noise along the optical fiber and the SNR improvement from 10.4 dB to 42.2 dB. The proposed demodulation algorithm is suitable for the detection of vibration signals with characteristic frequencies in the application of acoustic fault diagnosis for electromechanical devices.

## 1. Introduction

Vibration signal detection is critical for fault diagnosis of electromechanical devices, such as railway roller bearings [1] and internal combustion engines [2]. Owing to the ability of distributed sensing and transmission [3], anti-electromagnetic interference [4], and convenient installation, the optical fiber vibration sensor has grown significantly and has great potential in the field of fault diagnosis [5]. In practical application, because the position information and time-frequency characteristics of vibration signal need to be obtained simultaneously, the optical fiber vibration sensor based on coherent optical time domain reflectometry (COTDR) becomes an important choice, which is suitable for long-distance vibration positioning [6] and vibration phase recovery [7].

The electromechanical devices usually work in a noisy environment, which results in the difficult identification for characteristic frequency components of device fault [8]. Therefore, the COTDR sensing system needs to improve effectively its signal-to-noise ratio (SNR) to increase the detection sensitivity of the vibration signal. In recent years, many researchers study different methods for the improvement of SNR for COTDR sensing system, which could be summarized by the following two aspects: signal enhancement and noise elimination.

On the one hand, the signal enhancement of COTDR sensing system mainly relies on quality improvement of sensing pulsed light [9,10]. The effective modulation methods for sensing light have been research hotspots to improve the SNR. A variety of optical pulse modulation schemes are thus successively applied to generate sensing pulsed light with high extinction ratio (ER), such as: acousto-optic modulator (AOM) [11,12], electro-optic modulator (EOM) [13,14], two-level EOM [15]. However, ER values achieved by AOM and EOM are limited. So the research of optical pulse modulation is of practical importance to the signal enhancement.

On the other hand, the noise elimination is mainly dependent on a signal processing algorithm in time domain and frequency domain [16,17,18,19]. A fast Fourier transform is performed on the original signal matrix to separate the noise and vibration signals in the frequency domain to obtain a high SNR for the vibration location [16]. An active frequency scanning method is combined with a cross-correlation algorithm to dynamically reduce the noise caused by frequency drift [17]. A multi-scale wavelet decomposition method is used to decompose the time signal by the selective rearrangement in order to improve the SNR [18]. Therefore, an effective denoising algorithm of the COTDR sensing system has significant practical demands for the fault diagnosis of electromechanical devices.

As mentioned above, the vibration information of device faults to be detected has normally characteristic frequency components in the strong background noise environment. A cascade structure of AOM and a semiconductor optical amplifier (SOA) is firstly applied in this paper, because the SOA is a current-sensitive device which can generate stable pulsed light with a high ER [20]. The function of AOM in this structure is to offer the frequency shifting for the heterodyne detection in a COTDR sensing system. Moreover, the least mean square error (LMS) algorithm is adopted for the signal denoising. The LMS algorithm has the advantages of signal reduction and stability [21], it is thus suitable to realize the target vibration signal extraction in the strong background noise environment. In order to realize the LMS adoptive filter, the envelope demodulation algorithm [22] is applied on the processing of amplitude signal to obtain the reference frequency information of the vibration signal.

## 2. High ER System Based on the Cascade Structure

**Figure 1:**
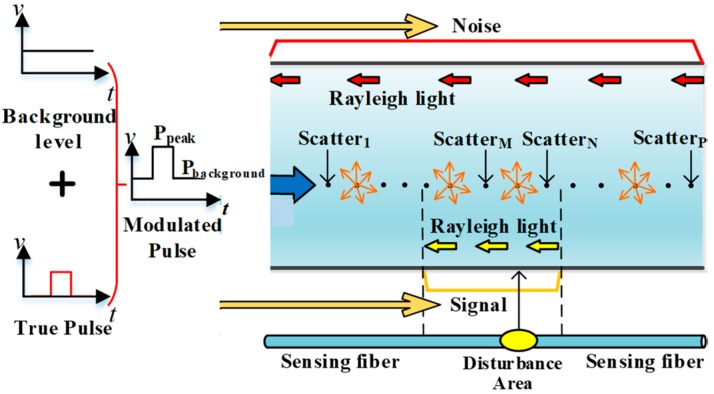
Theoretical model diagram of the influence of extinction ratio (ER) on signal-to-noise ratio (SNR).

In the COTDR sensing system, continuous light is modulated into pulsed light. The ER value of modulation devices could affect the detection sensitivity of the sensing system. As shown in Figure 1, the modulated light is composed of the background level light and the effective pulsed light. Considering the optical fiber as a series of scatters along the direction of optical fiber, the background level light could excite and superpose the backscattered Rayleigh light at every scatter. It cannot represent the characteristics of the backscattered Rayleigh light at a certain scatter, corresponding to the vibration position, and turns into noise finally. The effective pulsed light only excites backscattered Rayleigh light at the scatters within the pulse range, which can reflect the information of the vibration signal. In fact, multi-point vibrations could be monitored by the COTDR sensing system. The external vibration signal could affect any scatter of the optical fiber, so the coherent light within the pulse range could be modulated by the external vibration at any position of the optical fiber. Therefore, the ER value of the modulation device is determined by the background level power value of optical pulse and the peak power value of optical pulse, which can be expressed as *ER* = *P*_peak_/*P*_background_.

**Figure 2:**
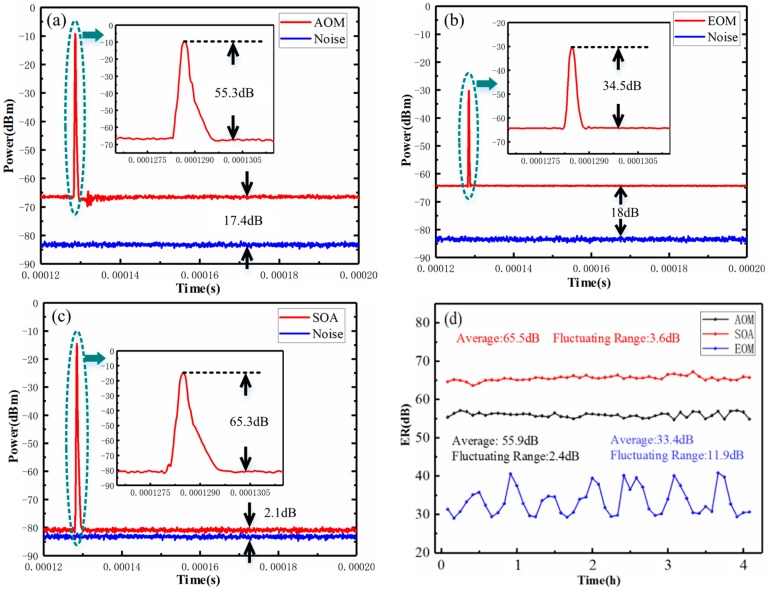
ER measurement results of (**a**) acousto-optic modulator (AOM), (**b**) electro-optic modulator (EOM), and (**c**) semiconductor optical amplifier (SOA). (**d**) ER stability evaluation results of AOM, SOA, and EOM.

According to earlier reports [20,23], the SOA device can obtain the higher ER value with good stability. Figure 2 shows the experimental verification results of ER value for different modulation devices, which are obtained by the spectrum analyzer. As shown in Figure 2a–c, the blue curves are the noise floor of the spectrum analyzer, which are about −82 dBm. The background level power value of optical pulses (*P*_background_) are about −64.6 dBm in Figure 2a, −64 dBm in Figure 2b, and −79.9 dBm in Figure 2c, which means that the SOA has the least leakage light. Moreover, the peak power value of the optical pulse (*P*_peak_) are −9.3 dBm in Figure 2a, −30.1 dBm in Figure 2b, and −14.6 dBm in Figure 2c. Therefore, the ER value of SOA, AOM, and EOM can be calculated to be 65.3 dB, 55.3 dB, and 34.5 dB, respectively.

In addition, the ER stability of three modulation devices is measured. The measurement time is set to be 4 h, and the experimental data is recorded every 5 min to obtain the stability curves. Figure 2d shows that the average ER value of SOA is about 65.5 dB and the fluctuating range is 3.6 dB. The average ER value of AOM is about 55.9 dB and the fluctuating range is 2.4 dB. The average ER value of EOM controlled by the bias control panel is about 33.4 dB and the fluctuating range is 11.9 dB. The experimental results show that the pulse modulation scheme based on SOA can obtain the highest ER with good stability, thus it is suitable for the COTDR sensing system with strong background noise in this paper.

**Figure 3:**
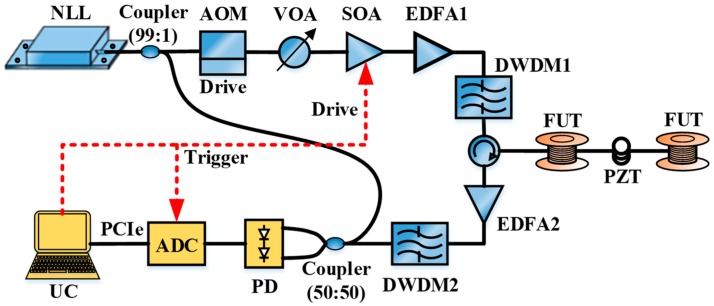
Experimental setup of coherent optical time domain reflectometry (COTDR) system.

Figure 3 shows the experimental setup of the COTDR sensing system. The ultra-narrow linewidth laser (NLL) emits continuous light with a linewidth of 100 Hz and a center wavelength of 1550.12 nm. It is split into two paths by a 1:99 coupler. The 1% path is used as the reference light for heterodyne detection, and the 99% path is used as signal light to be modulated by the cascade structure composed by the SOA and AOM.

The modulated optical frequency is shifted by 200 MHz based on the original light, and the AOM from the product of Gooch and Housego is driven by the direct current (DC) power source with a voltage of 3.3 V. The variable optical attenuator (VOA) achieves appropriate power attenuation for continuous light to meet the input power requirements of −6 dBm for the SOA. The SOA could then modulate the continuous light into the pulsed light with a repetition rate of 8 kHz and a pulse width of 200 ns.

The pulsed light is amplified by an erbium-doped fiber amplifier (EDFA1), and filtered by a dense wavelength division multiplexer (DWDM1). The pulsed light is incident into the sensing fiber by the circulator. The Rayleigh backscattered light including the vibration information is then returned from the circulator, amplified by EDFA2, and filtered by the DWDM2 to remove the useless frequency component. Finally, the amplified Rayleigh backscattered light beats with the reference light, the beat signal is detected by a balanced photo-detector (PD).

After photoelectric conversion, the data is collected and transmitted to the upper computer (UC) for the signal analysis and processing. The sampling rate of data acquisition is 1000 Msps. The length of sensing fiber under test (FUT) is 2000 m. The piezoelectric transducer (PZT) module is used to generate the vibration signal, which is driven by an arbitrary signal generator (AWG) and placed 1001 m away from the input end of sensing fiber. The winding length of optical fiber around the PZT module is 1.5 m.

## 3. Coherent Optical Time Domain Reflectometry (COTDR) System Based on Least Mean Square Error (LMS) Algorithm

### 3.1. Signal Demodulation for COTDR System

**Figure 4:**
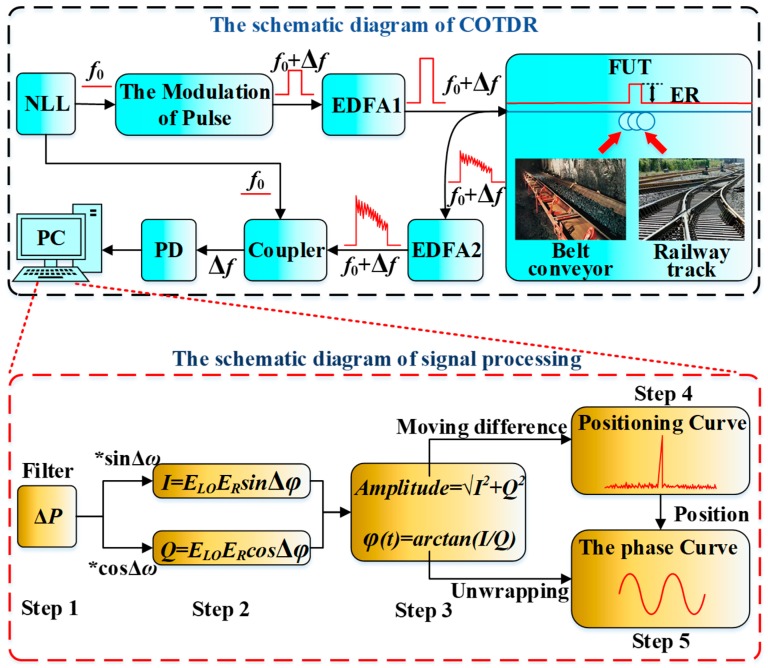
Signal demodulation schematic diagram of COTDR system.

The signal demodulation principle of the COTDR system is shown in Figure 4. The ultra-narrow linewidth laser (NLL) emits continuous light with a center frequency of *f*_0_, which is split into two paths. One path is used as the reference light for heterodyne detection, and the other path is used as sensing light which need to be modulated into pulsed light.

The conventional modulation unit is the AOM which could also realize the frequency shift with a fixed value Δ*f*. The parameters of optical pulse are the key factors for evaluating the sensing performance. The repetition rate could affect the sensing distance and the signal’s bandwidth, and the pulse width could affect the spatial resolution (SR) of the system. In addition, the performance of ER of the modulation unit could greatly affect the incident optical power and thus the SNR of the sensing system.

The phase and amplitude of the vibration signal is normally obtained by the In-phase and Quadrature (IQ) demodulation algorithm, which could reduce interference caused by external environmental changes.

The detected electronic power of the beat signal is *P_BS_*(*t*), which is given as follows:

(1) $$P_{BS}\left(t\right)=4kE_{R}E_{LO}\cos\left(\Delta \omega t+\varphi (t)\right)$$

where *E_R_* is the amplitude of Rayleigh backscattered light, *E_LO_* is the amplitude of reference light, *k* is the photoelectric conversion coefficient, Δ*ω* is the frequency shift introduced by AOM, *φ*(*t*) is the phase difference between reference light and sensing light.

Figure 4 shows also the schematic diagram of the IQ demodulation process. The first step is to retain the effective vibration information which is modulated at the carrier frequency Δ*ω*, by a finite impulse response (FIR) bandpass filter.

In step 2, the filtered signal is divided into two paths, which are respectively multiplied by a standard frequency shift (Δ*ω*) sine and cosine signal. Therefore, two components including double frequency and zero frequency are obtained, and the zero frequency component is retained. The two orthogonal components *I*(*t*) and *Q*(*t*) carrying the amplitude and phase of light intensity signal are given by the following equations [24]:

(2) $$I\left(t\right)=E_{R}E_{LO}\cos\left(\varphi \left(t\right)\right)$$

(3) $$Q\left(t\right)=E_{R}E_{LO}\sin\left(\varphi \left(t\right)\right)$$

In step 3, the amplitude of light intensity *A_LI_*(*t*) and the phase of light intensity *Φ_LI_*(*t*) are obtained by the trigonometric function’s operation [25], which are given as follows:

(4) $$A_{LI}\left(t\right)=\sqrt{I{\left(t\right)}^{2}+Q{\left(t\right)}^{2}}$$

(5) $$\Phi_{LI}\left(t\right)=\arctan\left(\frac{I(t)}{Q(t)}\right)$$

In step 4, because one optical pulse corresponds to one scattering curve, the amplitude of light intensity *A_LI_*(*t*) could be considered as a series of amplitude curves of single pulsed light. These amplitude curves of single pulsed light could form an time-space amplitude matrix *A_LI_*(*l*,Δ*t*), where the length of optical fiber *l* = (*ct*)/(2*n_f_*), *c* is the speed of light in vacuum, *n_f_* is the refractive index of optical fiber, Δ*t* is the time interval between two adjacent optical pulses. Therefore, the positioning curve is obtained by a moving differential algorithm [26], and the vibration position can be located. There will be obvious peaks at the vibration positions in the positioning curve. Suppose *A_i_*(*l*) is the *i*-th of the *N* amplitude curves, the vibration point will be evidently shown in the positioning curve *P*(*l*), which is expressed by:

(6) $$P\left(l\right)=\sum_{i=\Delta s+1}^{N}\frac{A_{i}\left(l\right)-A_{i-\Delta s}\left(l\right)}{A_{i}\left(l\right)}$$

where the Δ*s* is the curve step. For example, if the curve step Δ*s* is 3, it means that the first amplitude curve will be subtracted from the forth amplitude curve, the second amplitude curve will be subtracted from the fifth amplitude curve, and so on. Finally, all the results of subtraction need to be normalized and cumulatively averaged.

In step 5, the phase of light intensity *Φ_LI_*(*t*) could be considered as a series of phase curves of single pulsed light. These phase curves of single pulsed light could also form an time-space phase matrix *Φ_LI_*(*l*,Δ*t*). According to the vibration position obtained in step 4, the phase curve at the vibration position could be obtained along the time axis by the phase unwrapping process, which can be used to characterize the vibration signal generated by the device fault.

### 3.2. Adaptive Filtering Based on LMS Algorithm

However, because the environmental noise has a prominent effect on the detection performance, the vibration signal with characteristic frequency components is difficult to identify directly by the conventional IQ demodulation process. So the adaptive filtering based on LMS algorithm is applied in the COTDR sensing system to filter out the complex environmental noise.

**Figure 5:**
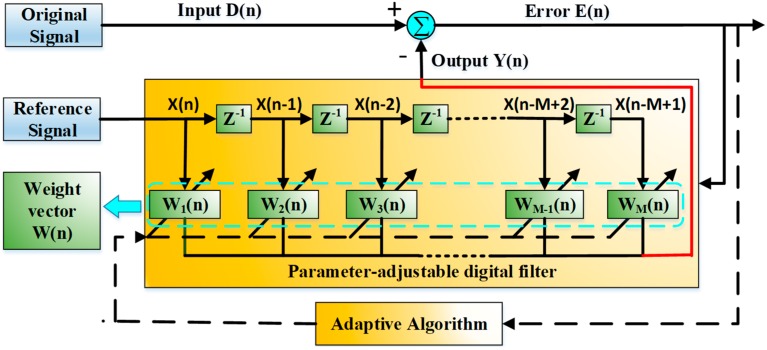
Schematic diagram of adaptive filtering based on least mean square error (LMS) algorithm.

Figure 5 shows the schematic diagram of adaptive filtering based on the LMS algorithm. The phase curve obtained in the step 5 of IQ demodulation process could be original signal, and then considered as *D*(*n*) after the discretization process. The input signal *D*(*n*) is processed by a parameter-adjustable digital filter. The reference signal *X*(*n*) of the filter is given by the prior time-frequency analysis of the vibration signal. The output signal *Y*(*n*) of the filter is approximated as possible closed to the reference signal by means of the iteration of weight vector *W*(*n*), which results from reference signal *X*(*n*) via the comparison with the error signal *E*(*n*). The weight vector *W*(*n*) and the output signal *Y*(*n*) are given as follows [27]:

(7) W(n+1)=W(n)+βE(n)X(n)

(8) $$Y\left(n\right)=W^{T}\left(n\right)X\left(n\right)$$

where *β* is the iterative step size of the weight vector. For proper stability, convergence speed and signal tracking capability of the filter, the step size needs to be constantly adjusted. Theoretically, a signal waveform highly similar to the reference signal could be obtained after LMS filtering.

### 3.3. Prior Time-Frequency Analysis for the Construction of Reference Signal

In the design process of LMS adaptive filter, the prior statistical characteristics of the reference signal could be given by the prior time-frequency analysis of the vibration signal. Because the vibration signal generated by the device fault is not an irregular random signal, it contains a series of characteristic components. Based on these characteristic components, we can construct the reference signal.

According to the principle of Fourier series, the reference signal *X*(*n*) can be superimposed by a series of characteristic components, which are sine waves of different harmonics, shown as follows:

(9) $$X(n)=\sum_{i=1}^{N}A_{i}\sin\left(2\pi f_{i}n\right)$$

where *N* is an integer greater than 1, *i* is the index of Fourier series, *A_i_* is the amplitude of *i*-th characteristic component, *f*_i_ is the frequency of *i*-th characteristic component. So we should carry out the prior time-frequency analysis of vibration signal in order to obtain these characteristic components *A_i_*sin(2π*f_i_*n). The prior time-frequency analysis means that a small part of beat signal *P_BS_*(*t*) in Equation (1) should be pre-processed for the construction of reference signal. After the reference signal is obtained, the rest of beat signal could be demodulated and filtered by the constructed LMS adaptive filter.

**Figure 6:**
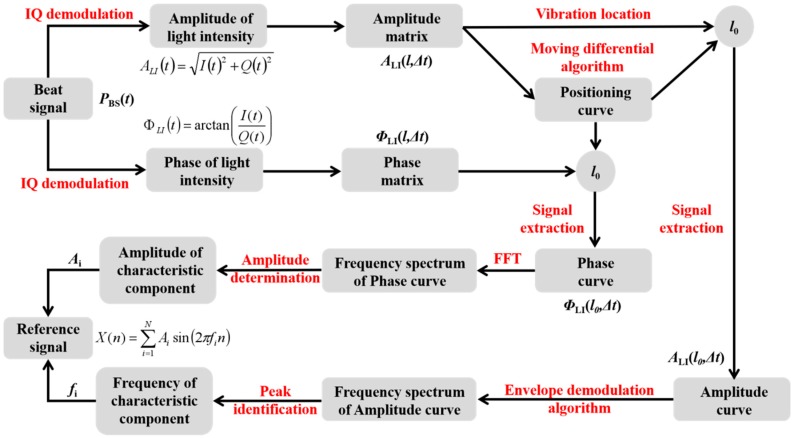
Schematic diagram of prior time-frequency analysis for construction of reference signal.

In order to obtain the frequency of characteristic component *f*_i_, and the amplitude of characteristic component *A_i_*, the schematic diagram of prior time-frequency analysis for the construction of reference signal is shown in Figure 6.

Firstly, we use the conventional IQ demodulation method to process the small part of beat signal, and obtain the amplitude of light intensity *A_LI_*(*t*) from the Equation (4) and the phase of light intensity *Φ_LI_*(*t*) from Equation (5). According to the time interval between two adjacent optical pulses Δ*t*, we could construct the time-space amplitude matrix *A_LI_*(*l*,Δ*t*) and the time-space phase matrix *Φ_LI_*(*l*,Δ*t*). 

Secondly, the positioning curve *P*(*l*) could be obtained after the processing step 4 of Figure 4 by using the moving differential algorithm. Supposing only one vibration signal is applied at the optical fiber, the vibration position could be located at the peak position *l*_0_. Therefore, we can carry out the signal extraction along the time axis. The amplitude curve *A_LI_*(*l*_0_,Δ*t*) at the vibration position *l*_0_ and the phase curve *Φ_LI_*(*l*_0_,Δ*t*) at the vibration position *l*_0_ could be obtained.

Thirdly, according to the envelope demodulation algorithm proposed by our group [22], the noise reduction could be applied for the amplitude curve without the loss of vibration frequency information. The DC component of amplitude curve *A_LI_*(*l*_0_,Δ*t*) is removed and the alternating current (AC) component of amplitude curve *A_LI_*(*l*_0_,Δ*t*) is unitized. So most of the noise could be eliminated and the vibration frequency information could be preserved. But due to the processing of envelope extraction, the amplitude information of a characteristic component will be also removed and result in inconsistency with the vibration signal. Therefore, after the envelope demodulation algorithm, the frequency spectrum of amplitude curve *A_LI_*(*l*_0_,Δ*t*) could be obtained. With the help of peak identification, all the frequencies of characteristic component *f_i_* could be obtained.

Finally, although the phase curve has a lot of noise, we can obtain the frequency spectrum of the phase curve by the Fast Fourier Transform (FFT) method. Because the phase curve has the linear relationship with vibration signal, we can carry out the amplitude determination at the obtained frequencies *f_i_*_._ and obtain all the amplitudes of characteristic component *A_i_*. Based on the obtained amplitudes of characteristic component *A_i_* and the obtained frequencies of characteristic component *f_i_*, we can construct the reference signal from Equation (9).

## 4. Results and Discussion

In order to evaluating the performance of LMS adaptive filtering for the detection of vibration signal, some experiments based on the COTDR system are performed with strong background noise. The vibration signal is simulated by the PZT module, which is driven by an AWG to generate the arbitrary signal. Therefore, a three-order sinusoidal harmonic signal *s* (*s* = 10·sin(2π*ft*) + 7·sin(4π*ft*) + 5·sin(6π*ft*), with *f* = 500 Hz) is programmed and generated by the AWG, with the frequencies of 500 Hz, 1000 Hz, and 1500 Hz. The corresponding peak-to-peak amplitudes are 10 V, 7 V, and 5 V, respectively.

**Figure 7:**
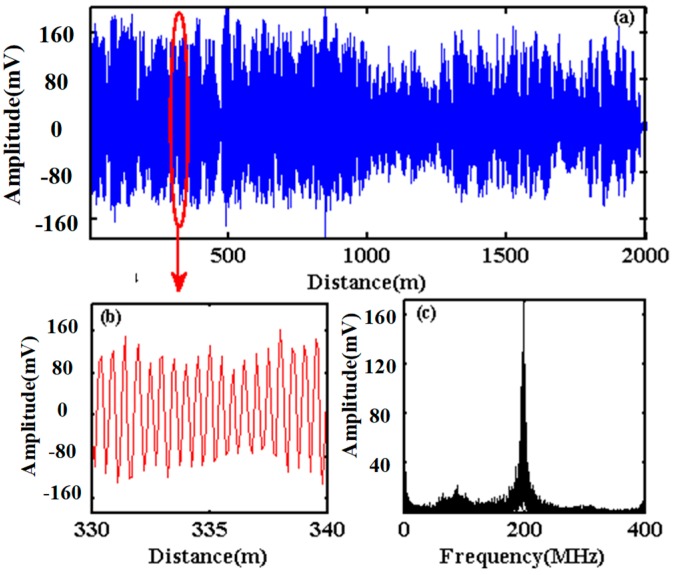
(**a**) Original coherence curve, (**b**) partial enlargement and (**c**) frequency spectrum of original coherence curve.

Figure 7a shows one of the original coherence curves of beat signal detected by the PD, along the optical fiber from 0 m to 2000 m. Figure 7b shows the enlargement of the original coherence curve between 330 m and 340 m. A quasi sinusoidal waveform is plotted, and the amplitude fluctuation is related with the interference of Rayleigh backscattered light within the optical pulse. Figure 7c shows the frequency spectrum of the original coherence curve, and a marked peak at 200 MHz is observed which corresponds to the frequency shift of 200 MHz generated by AOM.

Because the vibration information is modulated around 200 MHz, the original coherence curve could be filtered by a FIR band-pass filter with the cut-off frequencies at 150 MHz and 250 MHz. The process of IQ demodulation is then realized to obtain the amplitude curve and the phase curve, according to the schematic diagram in Figure 4.

**Figure 8:**
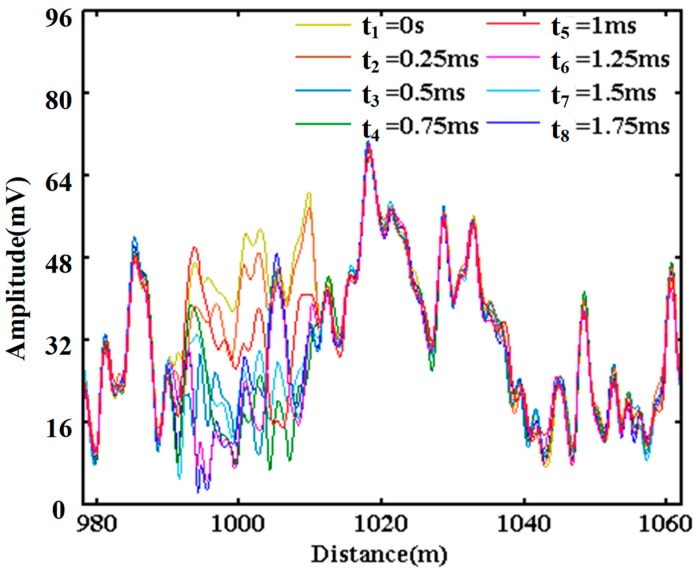
Superimposed amplitude curves of COTDR at different times.

Figure 8 shows the demodulated amplitude curves between 978 m and 1062 m, and these eight superimposed curves are chosen at different times between the instant *t*_1_ = 0 s and the instant *t*_8_ = 1.75 ms with a time step of 0.25 ms. The amplitude curves at no vibration areas are stable and basically coincident with each other, while the amplitude curves at the vibration area nearby the position of 1000 m are obviously varying, which could reflect the disturbance caused by the vibration signal. This phenomenon exists in the whole optical fiber. When there are many vibrations acting on the optical fiber, there will be many varying areas.

The complete vibration information including the waveform and the location needs an accurate positioning process. The moving difference algorithm is thus taken by the subtraction between two adjacent amplitude curves with different curve steps in order to obtain the vibration position from Equation (6). In addition, the wavelet algorithm is used to denoise the positioning curve for the identification of vibration position.

**Figure 9:**
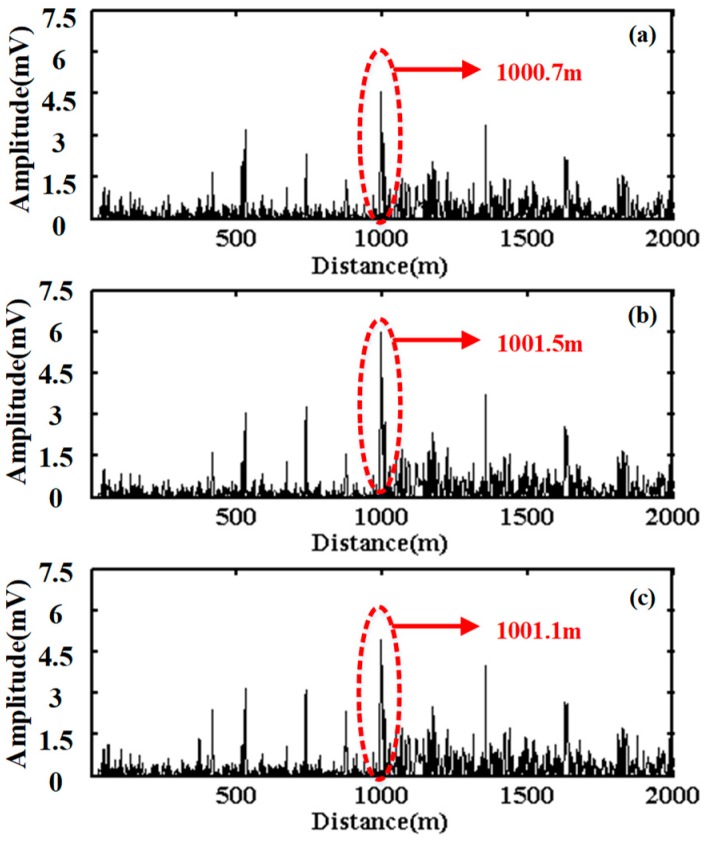
Positioning curves with the curve step of (**a**) 19, (**b**) 20, and (**c**) 21.

Figure 9 shows the positioning curves with different curve steps. The obvious peaks corresponding to the vibration position are obtained. The other peaks correspond to the noise signals, because the whole sensing fiber may be affected by the environmental noise. Here, two points in the positioning curve corresponding to the 10% of the peak value at the rising edge and the falling edge are chosen as the start and end of effective vibration area. So the middle value of these two points in the coordinate axis of distance is considered as the vibration position, which are 1000.7 m with the curve step of 19 in Figure 9a, 1001.5 m with the curve step of 20 in Figure 9b, and 1001.1 m with the curve step of 21 in Figure 9c. Moreover, in order to get a more accurate vibration position, the cumulative average of these three positioning values with different curve steps is performed, thus the final vibration position is chosen as 1001.1 m.

**Figure 10:**
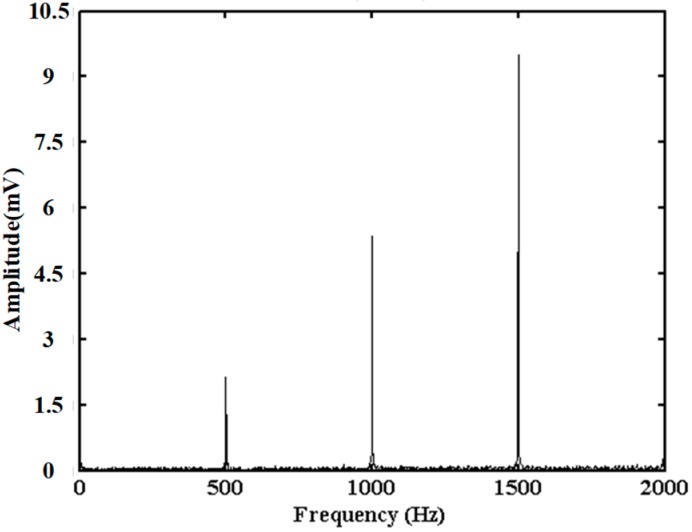
Frequency spectrum of amplitude curve after envelope demodulation algorithm.

Based on the construction schematic of LMS adaptive filter in Figure 6, the amplitude curve at the vibration position is extracted and processed by the envelope demodulation algorithm. Figure 10 shows the frequency spectrum of amplitude curve after the envelope demodulation algorithm. There are three peaks corresponding to the frequencies of 500 Hz, 1000 Hz, 1500 Hz, which are consistent with the frequencies of the applied vibration signal by PZT. According to these three frequencies obtained by the enveloped amplitude signal, the amplitude of above frequencies could be obtained in the FFT result of phase signal at vibration position, and could be used to generate the reference signal in the LMS adaptive filter.

**Figure 11:**
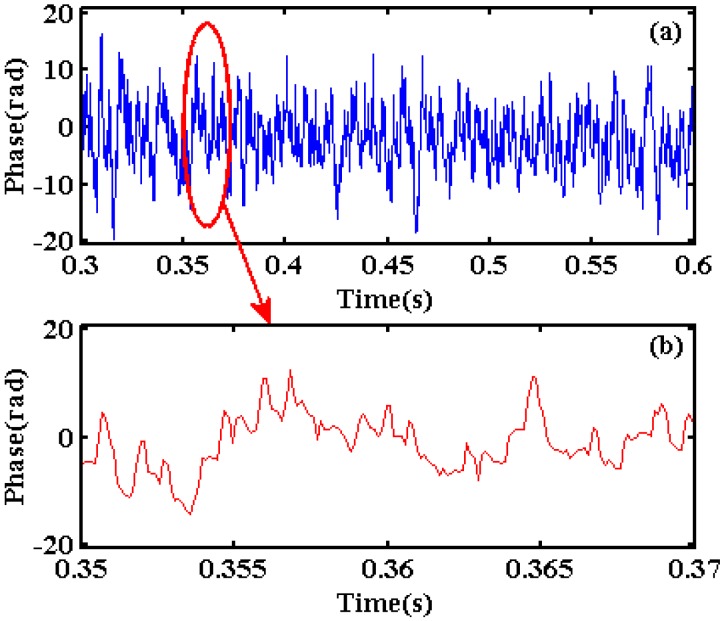
(**a**) Phase curve and (**b**) its partial enlargement at the vibration position.

According to the vibration position obtained by the moving difference algorithm, the phase curve at the vibration position of 1001.1 m could be extracted. Because the winding length of optical fiber is 1.5 m in the PZT module, the phase unwrapping process needs a subtraction between the phase curve at the vibration position of 1001.1 m and the phase curve at the no vibration area, which is separated from the vibration position by 40 points (4 m). This subtraction could eliminate the initial phase difference due to the time interval of different input optical pulses. The final phase curve between 0.3 s and 0.6 s is shown in Figure 11a and the partial enlargement of the phase curve between 0.35 s and 0.37 s is shown in Figure 11b. There are a lot of disturbed noises in the phase curve and the characteristic of three order sinusoidal harmonics could hardly be observed.

**Figure 12:**
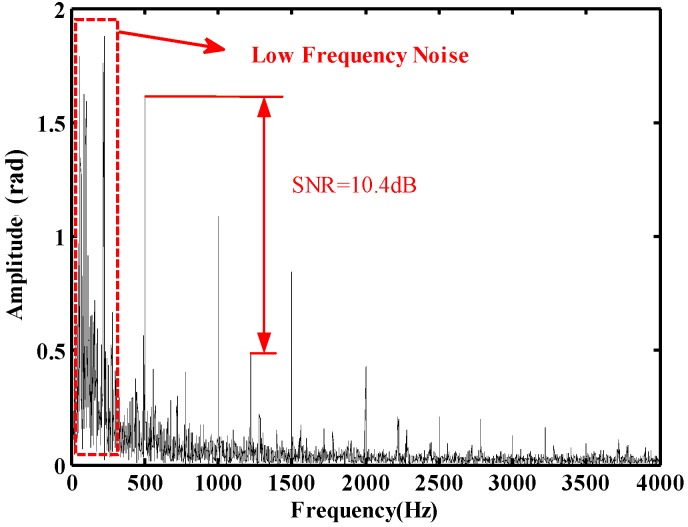
Frequency spectrum of phase curve.

Figure 12 shows the frequency spectrum of phase curve. An obvious noise distribution over all frequency ranges verifies the phenomena of disturbed noises in Figure 11. It is especially noted that there are more noise components in the low-frequency area (circled by a rectangle in red) up to 300 Hz, which lead to an inaccurate phase restoration. Moreover, the effective frequency components of 500 Hz, 1000 Hz and 1500 Hz are buried in the noise and the coefficient of 500 Hz, 1000 Hz and 1500 Hz is 10: 6.9: 5.2. The unexpected harmonics at 2000 Hz, 2500 Hz, and 3000 Hz are also visualized. Besides the complex and strong noise area in the low-frequency range, the SNR of the peak at 500 Hz in the frequency spectrum is only 10.4 dB.

Therefore, the LMS adaptive algorithm is needed to filter the noised phase curve. According to the three frequency components of 500 Hz, 1000 Hz, 1500 Hz, the corresponding amplitudes in the frequency spectrum of the phase curve is obtained. So the reference signal is generated to be *s*′ (*s*′ = 10·sin(2π*ft*) + 6.9·sin(4π*ft*) + 5.2·sin(6π*ft*), with *f* = 500 Hz). With the filter parameter optimization, the iterative step size of the weight vector *β* in Equation (7) is chosen to be 6 × 10^−6^, and the filter order is set to be 2000. After the dynamic acquisition of frequencies and amplitudes of vibration signals, the dynamic measuring of vibration signals in strong background noise based on the LMS algorithm can be achieved.

**Figure 13:**
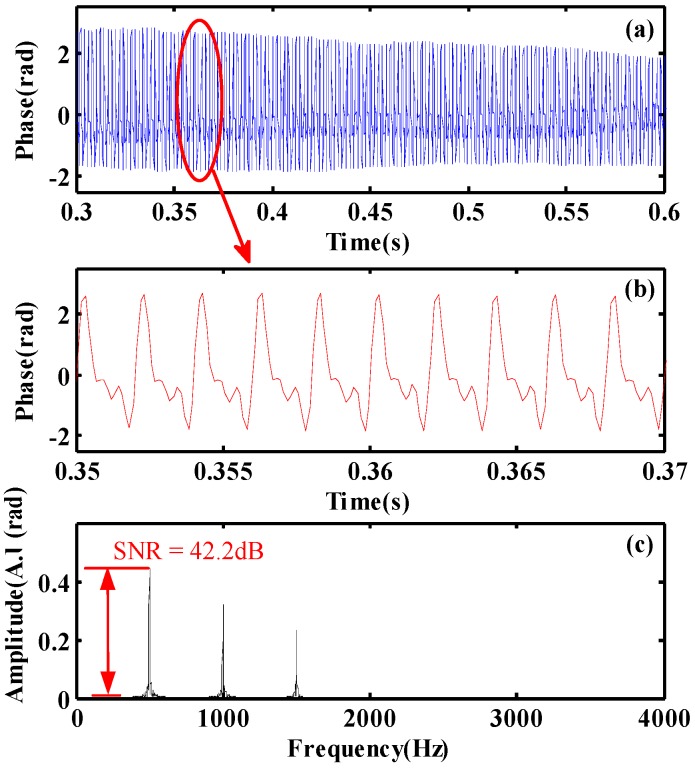
(**a**) Filtered phase curve and (**b**) its partial enlargement, (**c**) the frequency spectrum of the filtered phase curve.

The filtering result between 0.3 s and 0.6 s is shown in Figure 13a, and the amplitude distribution is regular and more stable. Figure 13b shows the filtered signal between 0.35 s and 0.37 s, which demonstrates the consistent characteristic of three order sinusoidal harmonic with the applied vibration signal. Figure 13c shows the frequency spectrum of the filtered phase curve. There are three frequency peaks at 500 Hz, 1000 Hz and 1500 Hz and the amplitude ratio is 10:7:5.2, which is approximately consistent with the amplitudes of the applied three-order sinusoidal harmonic vibration signal *s*. It proves that the extraction of vibration waveform is successful. In addition, the filtered signal obtains a SNR enhancement up to 42.2 dB at 500 Hz in the frequency spectrum.

**Figure 14:**
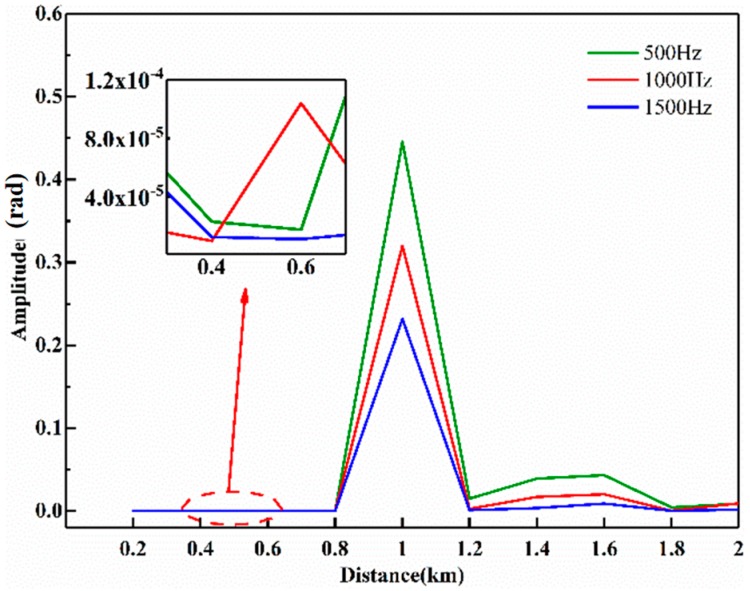
Amplitudes of 500 Hz, 1000 Hz and 1500 Hz in filtered phase curve along the optical fiber.

In order to further evaluate the effectiveness of the vibration signal restoration based on the LMS adaptive filtering algorithm, 10 phase curves are filtered by the LMS adaptive filter along the optical fiber length within the range of 200 m to 2000 m with the step of 200 m. The spectrum analysis is performed on the filtered results, and the frequency amplitudes of 500 Hz, 1000 Hz, and 1500 Hz in the spectrogram are separately recorded. Figure 14 shows three frequency components (500 Hz in green, 1000 Hz in red and 1500 Hz in blue) of filtered phase curves at different positions. As can be seen from the figure, three frequency components at the vibration position of 1001.1 m have the highest amplitudes and have a fixed proportional relationship corresponding to the applied vibration signal.

The amplitudes of three frequency components are largely weakened at no vibration positions. The thumbnail in the figure shows the amplification of amplitude curves from 350 m to 650 m marked by an ellipse in red, and the amplitudes of three frequency components are less than the level of 4 × 10^−5^, which means that there is huge magnitude difference as large as 10^4^ times. In addition, three frequency components do not have a linear relationship at no vibration positions. The spectrum analysis of filtered phase curves along the whole optical fiber proves that the LMS adaptive filtering algorithm can effectively extract the vibration signal in the strong noise environment.

## 5. Conclusions

In this paper, a distributed acoustic sensing system based on the cascade structure of AOM and SOA is proposed. Moreover, the phase signal is filtered by the LMS adaptive filter combined with prior time-frequency analysis. Thus, the dynamic phase change caused by the vibration can be extracted under strong noise. The SNR is improved from 10.4 dB to 42.2 dB. The proposed approach has great potential for the application of vibration signal detection during the fault diagnosis process of electromechanical devices in a strong noise environment.
